# *PITX3* DNA methylation is an independent predictor of overall survival in patients with head and neck squamous cell carcinoma

**DOI:** 10.1186/s13148-017-0317-7

**Published:** 2017-02-02

**Authors:** Verena Sailer, Emily Eva Holmes, Heidrun Gevensleben, Diane Goltz, Freya Dröge, Alina Franzen, Jörn Dietrich, Glen Kristiansen, Friedrich Bootz, Andreas Schröck, Dimo Dietrich

**Affiliations:** 1 Department of Pathology and Laboratory Medicine, Weill Cornell Medicine, New York, NY USA; 2 Caryl and Israel Englander Institute for Precision Medicine, Weill Cornell Medicine, New York, NY USA; 3 Institute of Pathology, University Hospital Bonn, Bonn, Germany; 4 Institute of Pathology, University Hospital Cologne, Cologne, Germany; 5 Department of Otorhinolaryngology, University Hospital Essen, University of Duisburg-Essen, Essen, Germany; 6 Department of Otolaryngology, Head and Neck Surgery, University Hospital Bonn, Sigmund-Freud-Str. 25, 53127 Bonn, Germany

**Keywords:** *PITX3*, Biomarker, Head and neck squamous cell carcinoma, HNSCC, DNA methylation, HPV, Prognosis

## Abstract

**Background:**

Molecular biomarkers assisting risk-group assignment and subsequent treatment stratification are urgently needed for patients with squamous cell cancer of the head and neck region (HNSCC). Aberrant methylation is a frequent event in cancer and, therefore, a promising source for potential biomarkers. Here, the methylation status of the paired-like homeodomain transcription factor 3 (*PITX3*) was evaluated in HNSCC.

**Methods:**

Using a quantitative real-time PCR, *PITX3* methylation was assessed in a cohort of 326 HNSCC patients treated for localized or locally advanced disease (training cohort). The results were validated with Infinium HumanMethylation450 BeadChip data from a 528 HNSCC patient cohort (validation cohort) generated by The Cancer Genome Atlas (TCGA) Research Network.

**Results:**

*PITX3* methylation was significantly higher methylated in tumor compared to normal adjacent tissue (NAT; training cohort: median methylation NAT 32.3%, tumor 71.8%, *p* < 0.001; validation cohort: median methylation NAT 16.9%, tumor 35.9%, *p* < 0.001). *PITX3* methylation was also significantly correlated with lymph node status both in the training (*p* = 0.006) and validation (*p* < 0.001) cohort. *PITX3* methylation was significantly higher in HPV-associated (p16-positive) tumors compared to p16-negative tumors (training cohort: 73.7 vs. 66.2%, *p* = 0.013; validation cohort: 40.0 vs. 33.1%, *p* = 0.015). Hypermethylation was significantly associated with the risk of death (training cohort: hazard ratio (HR) = 1.80, [95% confidence interval (CI) 1.20–2.69], *p* = 0.005; validation cohort: HR = 1.43, [95% CI 1.05–1.95], *p* = 0.022). In multivariate Cox analyses, *PITX3* added independent prognostic information. Messenger RNA (mRNA) expression analysis revealed an inverse correlation with *PITX3* methylation in the TCGA cohort.

**Conclusions:**

*PITX3* DNA methylation is an independent prognostic biomarker for overall survival in patients with HNSCC and might aid in the process of risk stratification for individualized treatment.

## Background

With an estimated incidence of more than 60,000 cases in the USA alone, cancer of the head and neck region is a common malignant disease. In male patients, cancer of the oral cavity and pharynx is the eighth most common cancer type. For 2016, 13,190 patients are estimated to succumb to the disease [1]. The most prevalent histological subtype in the head and neck region is squamous cell carcinoma (HNSCC), and common risk factors are the concurrent abuse of tobacco and alcohol as well as infections with a high-risk human papillomavirus (HPV) like HPV16 and HPV18. Depending on the causative agent, distinct differences have been recorded with regard to molecular alterations, tumor site, and prognosis of the tumors. While *TP53* mutations are the most common alterations in smoking-related cancers, HPV-associated tumors predominantly harbor *PIK3CA* mutations [2]. Moreover, patients with HPV-associated tumors tend to be younger, and tumors are more likely to develop in the oropharynx. Notably, these patients also have a better overall survival [3].

Most patients present in an advanced stage of disease. Surgery or radiation therapy with concurrent chemotherapy are first-line treatment options for HNSCC patients, but local and distant failure is common [4]. Newer drugs like the anti-EGFR antibody cetuximab have been successfully implemented into the therapeutic portfolio of HNSCC [5]. Combined with standard platinum-based chemotherapy, they provide a significant overall survival benefit for patients with recurrent or metastatic disease. For the same patient group, immunotherapy agents like the immune checkpoint inhibitor pembrolizumab have recently shown high efficacy [6]. Based on these results, pembrolizumab has recently been approved by the US Food and Drug Administration (FDA) for patients with recurrent or metastatic HNSCC. Considering the dismal prognosis in the event of metastatic or recurrent disease, such advances in drug therapy are urgently needed [7].

As yet, no prognostic molecular biomarkers have been established to assist clinicians in stratifying treatment or surveillance options after initial curative treatment. Currently, clinicopathological parameters like lymph node metastasis or tumor stage serve to identify high-risk patients. Additional biomarkers would be of utmost value to influence treatment strategy and surveillance intensity. With regard to emerging immunotherapy options that have an improved safety and side-effect profile, molecular biomarkers could distinguish patient subgroups that would benefit from adjuvant or neoadjuvant therapy. These biomarkers could further be evaluated in conjunction with other newly identified adverse risk factors like delayed time to treatment [8].

Tissue-based biomarkers have already been established for other tumor entities. In breast carcinoma, the commercially available multi-gene panel assay Oncotype DX^**©**^ estimates risk of recurrence after first-line treatment for early stage breast cancer by analyzing the expression of 21 genes [9]. Patients who have been identified as high risk based according to the subsequently calculated recurrence score may profit from intensified surveillance or adjuvant chemotherapy. Another promising biomarker approach is the investigation of the methylation status in a tumor sample. Epigenetic regulation is conferred through methylation, and malignant tumors are frequently hyper- or hypomethylated. Depending on the locus, methylation can result in gene transcription or repression [10]. Methylation analysis has already changed clinical practice for glioblastoma treatment, where promoter methylation of *MGMT* is predictive of response to the drug temozolomide [11]. So far, this is the only clinically implemented DNA methylation-based predictive biomarker test. DNA methylation can be quantified reliably and reproducibly via microarray, sequencing, or real-time PCR after bisulfite conversion of DNA [12–14]. Moreover, due to the stability of DNA methylation, the methylation status can be easily obtained even from degraded material like formalin-fixed paraffin-embedded (FFPE) tissue [15], which represents the most common clinically relevant sample material. Thus, studying epigenetic alterations is a promising approach for the development of biomarkers. Several epigenetic biomarkers have been tested in HNSCC, sometimes with conflicting results [16]. Hypermethylation of *TIMP3* and *CCNA1*, for instance, have been associated with an increased risk of developing second primary tumors [17]. Hypermethylation of a gene panel in salivary samples prior to treatment was shown to be prognostic of local recurrence and overall survival [18]. Consequently, epigenetic markers are emerging as prognostic tools in patients with HNSCC.

The paired-like homeodomain transcription factor 3 (*PITX3*), located on chromosome 10q24 [19], plays an important role in the midbrain dopamine system development, and single-nucleotide polymorphisms within the *PITX3* gene have been associated with Parkinson’s disease [20]. While the role of *PITX3* in the brain development has been well characterized, its contribution to tumorigenesis remains more elusive. It has been found to be hypermethylated in breast cancer tumors [21]. Recently, Holmes et al. reported that *PITX3* promoter methylation is strongly associated with biochemical recurrence-free survival in prostate cancer patients [22].

*PITX3* shares a sequence similarity and at least partly redundant function with the paired-like homeodomain 2 (*PITX2*) [21, 23]. *﻿PITX2* promoter methylation is a well studied prognostic biomarker in various cancers, i.e. prostate, breast, and bil﻿iary tract cancers ﻿[24–31]. Recent studies investigating the methylation status of *PITX2* in HNSCC and lung cancer cohorts found that hypermethylation was associated with improved survival [32, 33]. An in vitro diagnostic (IVD) test for anthracycline sensitivity in breast cancer patients based on *PITX2* methylation is currently being developed for commercial distribution by the company Therawis GmbH (Munich, Germany). Similar to *PITX2*, the closely related transcription factor *PITX1* plays a role in the embryonal development [34]. *PITX1* has been found to be predictive of chemosensitivity in a small HNSCC cohort [35].

Given the need for new prognostic biomarkers for patients with curatively treated HNSCC and the promising results regarding *PITX2* methylation as a biomarker, the methylation status of *PITX3* was investigated in well-annotated HNSCC cohorts.

## Methods

### Patients and ethics

The training cohort comprised 326 retrospectively enrolled HNSCC patients treated at the University Hospital Bonn. Clinical data were obtained for the majority of patients. The study was approved by the Institutional Review Board of the University Hospital Bonn, which waived the need for written informed consent. The validation cohort consisted of 528 HNSCC patients analyzed by The Cancer Genome Atlas (TCGA) Research Network (http://cancergenome.nih.gov/). All experiments were conducted in accordance with the Helsinki Declaration of 1975.

### Sample preparation, DNA extraction, and bisulfite conversion

Freshly cut sections from FFPE blocks were stained with hematoxylin and eosin. The tumor area was annotated and macrodissected from unstained slides for subsequent methylation analysis. The process of DNA extraction and bisulfite conversion was performed using the innuCONVERT Bisulfite All-In-One Kit (Analytik Jena, Germany) as previously reported [36]. In order to assess HPV status, the surrogate marker p16 was evaluated by immunohistochemistry.

DNA and messenger RNA (mRNA) from samples from the validation cohort were prepared as described by the TCGA Research Network (www.cancergenome.nih.gov).

### *PITX3* quantitative methylation analysis

For the training cohort, quantitative *PITX3* methylation analysis was performed by means of quantitative real-time PCR (qPCR) with primers and probes as previously described [22]. The analytical performance of the qPCR assay has been characterized earlier [22].

**Fig. 1:**
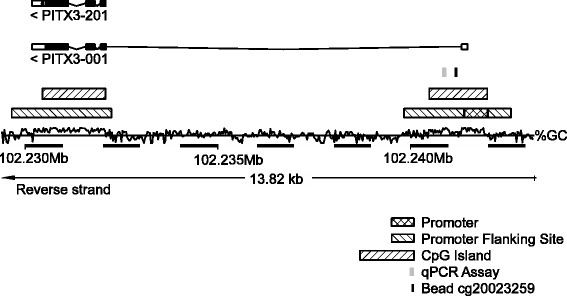
Genomic organization and assay position. Genomic organization of the *PITX3* gene and locations of the *PITX3* qPCR assay [22] and the Infinium HumanMethylation450 BeadChip bead cg20023259. The information was obtained from Ensembl Homo sapiens version GRCh38.p7

For the validation cohort, DNA methylation data were generated by the TCGA Research Network using the Infinium HumanMethylation450 BeadChip (Illumina, Inc., San Diego, CA, USA). HumanMethylation450 data of level 2 were downloaded directly from the TCGA webpage. The data files included background-corrected methylated (bead_M) and unmethylated (bead_U) bead pair summary intensities as extracted by the R package ‘methylumi’. The bead pair (cg20023259) in proximity to the locus of the qPCR assay was selected. Methylation values for the bead pair were calculated by the formula 100% × bead_M/(bead_M + bead_U). The position of the qPCR assay and the bead pair cg20023259 within the *PITX3* gene is shown in Fig. 1.

### *PITX3* mRNA expression analysis

The PITX3 mRNA data obtained from the TCGA Research Network were generated by means of the Illumina HiSeq 2000 RNA Sequencing version 2 analysis (Illumina, Inc., San Diego, CA, USA) as described by the TCGA Research Network (www.cancergenome.nih.gov). PITX3 mRNA expression (normalized counts) data of level 3 were downloaded from the TCGA webpage.

### Statistical analysis

Statistical analyses were performed using SPSS, version 24 (SPSS Inc., Chicago, IL, USA). Bivariate correlations were tested using the Spearman rank correlation coefficient (ρ). Overall survival analyses were conducted by Kaplan-Meier and Cox proportional hazards regression analyses. The Mann-Whitney *U* test and one-way analysis of variance (one-way ANOVA) were employed for the comparison of groups. *p* values <0.05 were considered significant.

## Results

### *PITX3* DNA methylation and mRNA expression in HNSCC tissue

**Fig. 2:**
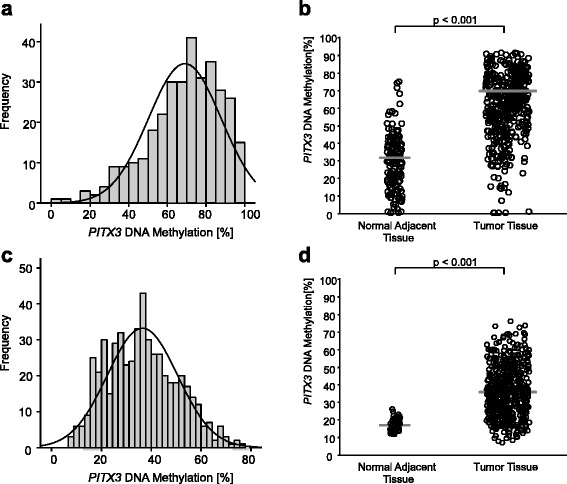
*PITX3* DNA methylation in HNSCC specimens. Frequency of *PITX3* DNA methylation in the training cohort (**a**) comprising 326 HNSCC patients and validation cohort (**b**) including 528 HNSCC patients. Shown are the methylation levels in tumor tissue only. *PITX3* DNA methylation in tumor tissue compared to normal adjacent tissue from the training (**c**) and the validation (**d**) cohort, respectively. Tumor tissue was methylated significantly higher compared to corresponding normal adjacent tissue. *p* values refer to Wilcoxon-Mann-Whitney test

The results from the validation cohort are entirely based upon data generated by the TCGA Research Network. The *PITX3* locus was highly methylated in tumor tissues from the training and the validation cohort. Histograms of *PITX3* methylation frequencies in both cohorts are shown in Fig. 2a, c. In the training cohort, methylation data were available for 466 patient samples including 140 normal and 326 tumor tissue samples. The comparison of *PITX3* methylation in tumor and normal adjacent tissue (NAT) revealed a significant difference between tumor tissue (mean 69.0%, median 71.8%) and NAT (mean 32.1%, median 32.3%; *p* < 0.001; Fig. 2b). Methylation ranged from 0.79 to 100% in tumor tissue and from 0 to 82.1% in normal tissue. This finding was confirmed in the validation cohort, in which methylation data were available for 578 patient samples including 50 normal and 528 tumor tissue samples. Methylation was significantly different between tissue types (*p* < 0.001; Fig. 2d) and ranged from 6.9 to 76.3% in tumor tissue (mean 36.5%, median 35.9%) and from 11.7 to 26.1% in normal tissue (mean 17.1%, median 16.9%). The methylation differences observed between the two analyzed cohorts might be caused by the application of two different technologies: qPCR and Infinium HumanMethylation450K BeadChip.

Furthermore, DNA methylation was shown to be inversely correlated with *PITX3* mRNA (*p* = 0.036) in the validation cohort. Interestingly, mRNA levels were also significantly different in tumor (mean 6.55 normalized counts) compared to normal (mean 15.37 normalized counts) tissue (*p* < 0.001).

### Association of *PITX3* DNA methylation with clinico-pathological parameters

Both in the training and validation cohort, *PITX3* DNA methylation was significantly associated with lymph node status (*p* = 0.006 and *p* < 0.001, respectively). Moreover, a significant methylation difference was found in regard to HPV status in the training cohort. Using p16 as surrogate marker, p16-negative tumors were significantly lower methylated than p16-positive tumors (66.2 vs. 73.7%, *p* = 0.018). This was confirmed in the validation cohort: HPV-associated (p16-positive) tumors were significantly highly methylated than p16-negative tumors (40.0 vs. 33.1%*, p* = 0.015). In addition, a significant difference in methylation was found when analyzing tumor site in both cohorts (training cohort: *p* = 0.023, validation cohort: *p* = 0.017). A detailed analysis of both cohorts can be found in Table 1.

**Table 1:** Association of clinicopathological parameters with *PITX3* methylation in HNSCC patients of the training cohort (*n* = 326) and the validation cohort (*n* = 528)

	Training cohort			Validation cohort (TCGA)		
Variable	No. (%) of patients	Mean *PITX3* methylation (%)	*p* value	No. (%) of patients	Mean *PITX3* methylation (%)	*p* value
All patients	326 (100)	69.0		528 (100)	36.5	
Sex			0.58			0.42
Female	74 (22.7)	68.0		142 (26.9)	35.6	
Male	252 (77.3)	69.3		386 (73.1)	36.8	
Age (years)			0.16			0.32
Mean	62.2			60.9		
Median	62		0.55	61		0.48
*n* < Median	132 (40.5)	70.3		282 (53.4)	36.1	
*n* > Median	119 (36.5)	71.7		245 (46.4)	36.9	
Unknown	75 (23.0)			1 (0.2)		
Smoking status			0.91			0.92
Non-smoker	25 (7.7)	69.8		122 (23.1)	36.7	
Smoker	178 (54.6)	70.2		393 (74.4)	36.6	
Unknown	123 (37.7)			13 (2.5)		
Pack years			0.17			0.60
<40	92 (28.2)	72.0		168 (31.8)	36.5	
>40	46 (14.1)	76.0		130 (24.6)	37.3	
Unknown	188 (57.7)			230 (43.6)		
Alcohol consumption			0.10			0.37
Yes	81 (24.8)	74.8		352 (66.7)	37.0	
No	47 (14.4)	69.8		165 (31.3)	35.9	
Unknown	198 (60.7)			11 (2.1)		
Tumor site			0.023^a^			0.017^a^
Oral cavity	50 (15.3)	69.0		320 (60.6)	36.6	
Oropharynx	139 (42.6)	72.0		81 (15.3)	40.2	
Hypopharynx	22 (6.7)	72.8		10 (1.9)	33.6	
Larynx	95 (29.1)	67.0		117 (22.2)	33.9	
Unknown	20 (6.1)					
pT			0.80			0.22
T1/T2	160 (49.1)	69.8		190 (36.0)	34.9	
T3/T4	119 (36.5)	69.2		276 (52.3)	36.5	
Unknown	47 (14.4)			62 (11.7)		
pN			0.006^a^			<0.001^a^
N0	128 (39.3)	66.3		180 (34.1)	32.8	
N1/2/3	173 (53.1)	72.0		240 (45.5)	37.7	
Unknown	25 (7.7)			108 (20.5)		
p16			0.013^a^			0.015^a^
Negative	200 (61.3)	66.2		74 (14)	33.1	
Positive	51 (15.6)	73.7		41 (7.8)	40.0	
Unknown	75 (23.0)			413 (78.2)		
Grade			0.40			0.33
1	7 (1.8)	62.3		63 (11.9)	34.7	
2	199 (49.9)	70.1		311 (58.9)	36.3	
3	107 (26.8)	68.8		125 (23.7)	37.5	
4	0 (0)			7 (1.3)	43.5	
Unknown	86 (21.6)			22 (4.2)		
Surgical margin			0.18			0.40
Negative	225 (69.0)	69.7		407 (77.1)	36.0	
Positive	41 (12.6)	65.6		60 (11.4)	37.6	
Unknown	60 (18.4)			61 (11.6)		
Second tumor			0.070	*NA*		
Yes	38 (11.7)	72.7				
No	140 (42.9)	77.9				
Unknown	148 (45.4)			528 (100)		
Vascular invasion			0.083			0.072
Yes	22 (6.7)	63.0		124 (23.5)	38.4	
No	170 (52.1)	70.1		232 (43.9)	35.6	
Unknown	134 (41.1)			172 (32.6)		

Mann-Whitney *U* test for sex, age (dichotomized), smoking status, pT, p16, surgical margin, second tumor, lymphovascular invasion, and pack years; One-way ANOVA for alcohol consumption, lymph node, grade, and tumor site; Spearman’s rank correlation for age. Results from the validation cohort are entirely based upon the data generated by the TCGA Research Network: http://cancergenome.nih.gov/

*NA* data not available

^a^significance

### Association of *PITX3* DNA methylation with survival

**Fig. 3:**
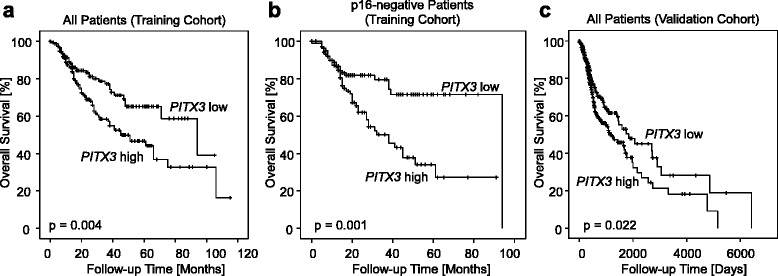
Overall survival in patients stratified according to *PITX3* DNA methylation. Kaplan-Meier analysis of overall survival in HNSCC patients stratified according to *PITX3* DNA methylation levels in tumor tissue. **a** Training cohort (all patients). **b** Training cohort (p16-negative patients). **c** Validation cohort (all patients). High *PITX3* DNA methylation was associated with an adverse prognosis in both cohorts, in particular in p16-negative patients from the training cohort. A subgroup analysis of p16-negative patients from the validation cohort was not performed due to largely missing data

Higher *PITX3* DNA methylation analyzed as continuous variable showed a strong trend towards poorer overall survival in the training cohort (Hazard ratio (HR) = 1.01, [95% confidence interval (95%CI) 1.00–1.02], *p* = 0.061). *PITX3* DNA methylation levels were subsequently dichotomized using an optimized cut-off (72.5%). *PITX3* DNA hypermethylation was significantly associated with an increased risk of disease-related death in univariate Cox proportional hazards (HR = 1.80, [95%CI 1.20–2.69], *p* = 0.005; Table 2) and Kaplan-Meier (*p* = 0.004; Fig. 3a) analyses. In addition, p16 expression and N category were significantly associated with survival (Table 2). Furthermore, vascular invasion (V) was significantly associated with poor overall survival (HR = 3.51, [95%CI 1.90–6.47], *p* < 0.001).

**Table 2:** Univariate and multivariate Cox proportional hazard analysis of overall survival of HNSCC patients in the training cohort (*n* = 307)

	Univariate		Multivariate	
Variable	HR (95%CI)	*p* value	HR (95%CI)	*p* value
*PITX3* methylation (dichotomized, optimized cut-off 72.5%)	1.80 (1.20–2.69)	0.005^a^	2.28 (1.29–4.00)	0.004^a^
p16 expression (neg. reference)	0.40 (0.20–0.81)	0.010^a^	0.27 (0.11–0.67)	0.005^a^
N category	1.28 (1.07–1.53)	0.008^a^	1.27 (0.97–1.67)	0.82
T category	1.14 (0.96–1.36)	0.14	1.33 (1.03–1.72)	0.029^a^

Nineteen patients from the 326 patients of the cohort were omitted from survival analysis due to missing follow-up data

*NA* not analyzed

^a^significant feature

Interestingly, when performing a subgroup analysis among patients with p16-negative tumors, patients with *PITX3* hypermethylated tumors revealed an HR of 2.44 ([95%CI 1.44–4.14], *p* = 0.001) compared to patients with hypomethylated cancers. This finding was confirmed in Kaplan-Meier analysis (*p* = 0.001; Fig. 3b). No significant survival differences were seen in *PITX3*-stratified patients with HPV-positive tumors. In a multivariate analysis, *PITX3* methylation status added independent prognostic information (HR = 2.28, [95%CI 1.29–4.00], *p* = 0.004; Table 2) about the risk of dying. Vascular invasion status was excluded from multivariate analysis due to largely missing data from both cohorts.

The prognostic biomarker performance was validated in the TCGA cohort. Data were dichotomized by means of an optimized cut-off (35.1%). Univariate analysis showed a significant risk of death for patients with hypermethylated tumors (HR = 1.43, [95%CI 1.05–1.95], *p* = 0.022). In a multivariate Cox proportional hazard analysis, *PITX3* methylation added significant prognostic information to T and N category for *PITX3* methylation as dichotomized variable (HR = 1.49, [95%CI 1.02–2.16], *p* = 0.038; Table 3). However, mRNA expression analyzed as a continuous variable did not reveal any prognostic significance (HR = 1.00, [95%CI 0.98–1.02]; *p* = 0.74). The introduction of an optimized cut-off (normalized counts: 13.52) classified 58 patients as PITX3 mRNA expression-positive and 462 patients as negative. Only a trend towards a better prognosis of expression-positive patients was found (HR = 0.59, [95%CI 0.34–1.03], *p* = 0.062).

**Table 3:** Univariate and multivariate Cox proportional hazard analysis of overall survival of HNSCC patients in the validation cohort from The Cancer Genome Atlas (*n* = 528)

	Univariate		Multivariate	
Variable	HR (95%CI)	*p* value	HR (95%CI)	*p* value
*PITX3* methylation (dichotomized, optimized cut-off 35.1%)	1.43 (1.05–1.95)	0.022^a^	1.49 (1.02–2.16)	0.038^a^
N category	1.39 (1.14–1.70)	0.001^a^	1.25 (1.02–1.54)	0.035^a^
T category	1.31 (1.12–1.54)	0.001^a^	1.28 (1.06–1.56)	0.013^a^

p16 expression was omitted from analysis due to largely missing data

*NA* not analyzed

^a^significant feature

## Discussion and conclusions

Squamous cell cancer of the head and neck region poses a substantial global health burden and affects patients with a history of nicotine and alcohol abuse as well as patients harboring oral infections with high-risk HPV. The disease poses a large health burden worldwide. Surgery and radiotherapy are the mainstay of therapy with curative intent. Recent advantages in reconstructive techniques may help to reduce morbidity after extensive surgery of radiation-induced damage [37–39]. For patients undergoing curative therapy, biomarkers are urgently needed to identify individuals who are likely to experience tumor recurrence or metastasis and subsequent disease-related death. These patients could benefit from intensified surveillance or adjuvant therapy. Moreover, new immunotherapeutic drugs are emerging from which high-risk patients might benefit.

Epigenetic alterations in form of hypo- or hypermethylated DNA are frequent events in cancer. Investigating the DNA methylation status in patients with malignant diseases is a promising approach to establish new biomarkers. In the present study, *PITX3* DNA methylation was analyzed in a well-annotated HNSCC cohort from the University Hospital Bonn. In multivariate analysis, *PITX3* DNA hypermethylation was significantly associated with the risk of death. These results were validated in a HNSCC cohort from the TCGA Research Network. Furthermore, hypermethylation correlated inversely with PITX3 mRNA expression in the TCGA cohort. Although these findings lead to the conclusion that hypermethylation is associated with silencing of the *PITX3* gene, the transcriptional relevance remains unclear. Hypomethylation may result in transcription of the gene; however, the role of *PITX3* in tumorigenesis has not been described. One possible mechanism could be the co-transcription of other genes on chromosome 10q such as suppressor of fused homolog (*SUFU*), which acts as a negative regulator of the hedgehog signaling pathway. Combined loss of *SUFU* and *p53* results in the development of medulloblastomas and rhabdomyosarcoma in mice [40]. Hedgehog signaling may also play a role in HNSCC tumorigenesis. Hence, the transcription of a negative regulator could result in a reduced tumor growth [41]. It has previously been shown that HPV infection may result in alteration of the DNA methylome. Therefore, HPV-mediated epigenetic alterations may account for the hypermethylation in HPV-positive tumors described in the present study [42, 43]. However, *SUFU* and *PITX3* are not located on the immediately neighboring regions of 10q24. It is therefore unclear whether methylation of *PITX3* could have any influence on *SUFU* transcription.

In a rat model for prostate cancer development following exposure to endocrine-disrupting chemicals, *PITX3* was found to be one of several hypomethylated and therefore expressed genes [44]. While this model cannot serve as a model for HNSCC tumorigenesis, it is an interesting finding and possibly allows for the implication of *PITX3* alteration being an early event in cancer development. This would be in line with the results of Holmes et al., who were able to show that *PITX3* methylation is a strong prognostic marker for biochemical recurrence in localized prostate cancer [22].

HNSCC display a high genomic instability with a mean of 141 copy number alterations per tumor, including amplification of 10q24 [45, 46]. Since amplification has been associated with hypermethylation, this might provide an explanation for the observed methylation differences [47]. Even though the biological function remains unclear for now, *PITX3* DNA methylation status emerges as a promising biomarker particularly for patients with HPV-negative tumors.

In brief, this is the first study to show that the methylation status of *PITX3* is an independent prognostic factor for overall survival in patients with HNSCC. These findings warrant further investigation in a prospective study setting.

## References

[1] Siegel RL, Miller KD, Jemal A (2016). Cancer statistics, 2016. CA Cancer J Clin.

[2] Cancer Genome Atlas Network. Comprehensive genomic characterization of head and neck squamous cell carcinomas. Nature. 2015;517(7536):576–82. 10.1038/nature14129 PMC4311405 25631445

[3] Dok R, Nuyts S. HPV positive head and neck cancers: molecular pathogenesis and evolving treatment strategies. Cancers. 2016;8(4):41. 10.3390/cancers8040041 PMC4846850 27043631

[4] Sacco AG, Cohen EE (2015). Current treatment options for recurrent or metastatic head and neck squamous cell carcinoma. J Clin Oncol.

[5] Vermorken JB, Mesia R, Rivera F, Remenar E, Kawecki A, Rottey S (2008). Platinum-based chemotherapy plus cetuximab in head and neck cancer. N Engl J Med.

[6] Chow LQ, Haddad R, Gupta S, Mahipal A, Mehra R, Tahara M, et al. Antitumor activity of pembrolizumab in biomarker-unselected patients with recurrent and/or metastatic head and neck squamous cell carcinoma: results from the phase Ib KEYNOTE-012 expansion cohort. J Clin Oncol. 2016;34(32):3838–45. 10.1200/JCO.2016.68.1478 PMC6804896 27646946

[7] Echarri MJ, Lopez-Martin A, Hitt R. Targeted therapy in locally advanced and recurrent/metastatic head and neck squamous cell carcinoma (LA-R/M HNSCC). Cancers. 2016;8(3):27. 10.3390/cancers8030027 PMC4810111 26927178

[8] Murphy CT, Galloway TJ, Handorf EA, Egleston BL, Wang LS, Mehra R (2016). Survival impact of increasing time to treatment initiation for patients with head and neck cancer in the United States. J Clin Oncol.

[9] Chau NG, Li YY, Jo VY, Rabinowits G, Lorch JH, Tishler RB, et al.Incorporation of next-generation sequencing into routine clinical care to direct treatment of head and neck squamous cell carcinoma. Clin Cancer Res. 2016;22(12):2939–49. 10.1158/1078-0432.CCR-15-2314 26763254

[10] Jones PA (2012). Functions of DNA methylation: islands, start sites, gene bodies and beyond. Nat Rev Genet.

[11] Seystahl K, Wick W, Weller M (2016). Therapeutic options in recurrent glioblastoma—An update. Crit Rev Oncol Hematol.

[12] Dietrich D, Hasinger O, Banez LL, Sun L, van Leenders GJ, Wheeler TM (2013). Development and clinical validation of a real-time PCR assay for PITX2 DNA methylation to predict prostate-specific antigen recurrence in prostate cancer patients following radical prostatectomy. J Mol Diagn.

[13] Schatz P, Dietrich D, Koenig T, Burger M, Lukas A, Fuhrmann I (2010). Development of a diagnostic microarray assay to assess the risk of recurrence of prostate cancer based on PITX2 DNA methylation. J Mol Diagn.

[14] Dietrich D. Direct quantitative bisulfite sequencing using tag-modified primers and internal normalization. Anticancer Res. 2016;36(12):6343–6. 10.21873/anticanres.11231 27919955

[15] Jung M, Uhl B, Kristiansen G, Dietrich D. Bisulfite conversion of DNA from tissues, cell lines, buffy coat, FFPE tissues, microdissected cells, swabs,sputum, aspirates, lavages, effusions, plasma, serum, and urine. Methods Mol Biol. 2015. [Epub ahead of print]. 10.1007/7651_2015_260 26138988

[16] Juodzbalys G, Kasradze D, Cicciu M, Sudeikis A, Banys L, Galindo-Moreno P, et al. Modern molecular biomarkers of head and neck cancer. Part I. epigenetic diagnostics and prognostics: systematic review. Cancer Biomark. 2016;17(4):487–502. 10.3233/CBM-160666 PMC13020507 27802200

[17] Rettori MM, de Carvalho AC, Longo AL, de Oliveira CZ, Kowalski LP, Carvalho AL (2013). TIMP3 and CCNA1 hypermethylation in HNSCC is associated with an increased incidence of second primary tumors. J Transl Med.

[18] Carvalho AL, Henrique R, Jeronimo C, Nayak CS, Reddy AN, Hoque MO (2011). Detection of promoter hypermethylation in salivary rinses as a biomarker for head and neck squamous cell carcinoma surveillance. Clin Cancer Res.

[19] Chen H, Song Z, Yuan L, Xiong W, Yang Z, Gong L, et al. Genetic analysis of PITX3 variants in patients with essential tremor. Acta Neurol Scand. 2016. [Epub ahead of print]. 10.1111/ane.12608 27145793

[20] Jimenez-Jimenez FJ, Garcia-Martin E, Alonso-Navarro H, Agundez JA (2014). PITX3 and risk for Parkinson’s disease: a systematic review and meta-analysis. Eur Neurol.

[21] Dietrich D, Lesche R, Tetzner R, Krispin M, Dietrich J, Haedicke W (2009). Analysis of DNA methylation of multiple genes in microdissected cells from formalin-fixed and paraffin-embedded tissues. J Histochem Cytochem.

[22] Holmes EE, Goltz D, Sailer V, Jung M, Meller S, Uhl B (2016). PITX3 promoter methylation is a prognostic biomarker for biochemical recurrence-free survival in prostate cancer patients after radical prostatectomy. Clin Epigenetics.

[23] L'Honore A, Coulon V, Marcil A, Lebel M, Lafrance-Vanasse J, Gage P (2007). Sequential expression and redundancy of Pitx2 and Pitx3 genes during muscle development. Dev Biol.

[24] Uhl B, Gevensleben H, Tolkach Y, Sailer V, Majores M, Jung M, et al. PITX2 DNA Methylation as Biomarker for Individualized Risk Assessment of Prostate Cancer in Core Biopsies. J Mol Diagn. 2017;19(1):107–14. 10.1016/j.jmoldx.2016.08.008 27939865

[25] Uhl B, Dietrich D, Branchi V, Semaan A, Schaefer P, Gevensleben H, et al. DNA Methylation of PITX2 and PANCR Is Prognostic for Overall Survival in Patients with Resected Adenocarcinomas of the Biliary Tract. PLoS One. 2016;11(10):e0165769. 10.1371/journal.pone.0165769 PMC5087948 27798672

[26] Vinarskaja A, Schulz WA, Ingenwerth M, Hader C, Arsov C. Association of PITX2 mRNA down-regulation in prostate cancer with promoter hypermethylation and poor prognosis. Urol Oncol. 2013;31(5):622–7. 10.1016/j.urolonc.2011.04.010 21803613

[27] Bañez LL, Sun L, van Leenders GJ, Wheeler TM, Bangma CH, Freedland SJ, et al. Multicenter Clinical Validation of PITX2 Methylation as a Prostate Specific Antigen Recurrence Predictor in Patients With Post-Radical Prostatectomy Prostate Cancer. J Urol. 2010;184(1):149–56. 10.1016/j.juro.2010.03.012 20478579

[28] Weiss G, Cottrell S, Distler J, Schatz P, Kristiansen G, Ittmann M, et al. DNA Methylation of the PITX2 Gene Promoter Region is a Strong Independent Prognostic Marker of Biochemical Recurrence in Patients With Prostate Cancer After Radical Prostatectomy. J Urol. 2009;181(4):1678–85. 10.1016/j.juro.2008.11.120 19233404

[29] Hartmann O, Spyratos F, Harbeck N, Dietrich D, Fassbender A, Schmitt M, et al. DNA Methylation Markers Predict Outcome in Node-Positive, Estrogen Receptor-Positive Breast Cancer with Adjuvant Anthracycline-Based Chemotherapy. Clin Cancer Res. 2009;15(1):315–23. 10.1158/1078-0432.CCR-08-0166 19118060

[30] Harbeck N, Nimmrich I, Hartmann A, Ross JS, Cufer T, Grützmann R, et al. Multicenter Study Using Paraffin-Embedded Tumor Tissue Testing PITX2 DNA Methylation As a Marker for Outcome Prediction in Tamoxifen-Treated, Node-Negative Breast Cancer Patients. J Clin Oncol. 2008;26(31):5036–42. 10.1200/JCO.2007.14.1697 18711169

[31] Nimmrich I, Sieuwerts AM, Meijer-van Gelder ME, Schwope I, Bolt-de Vries J, Harbeck N, et al. DNA hypermethylation of PITX2 is a marker of poor prognosis in untreated lymph node-negative hormone receptor-positive breast cancer patients. Breast Cancer Res Treat. 2008;111(3):429–37. 10.1007/s10549-007-9800-8 17965955

[32] Sailer V, Holmes EE, Gevensleben H, Goltz D, Droge F, de Vos L, et al. PITX2 and PANCR DNA methylation predicts overall survival in patients with head and neck squamous cell carcinoma. Oncotarget. 2016;7(46):75827-38. 10.18632/oncotarget.12417 PMC5342781 27716615

[33] Holmes EE, Jung M, Meller S, Leisse A, Sailer V, Zech J, et al. Performance Evaluation of Kits for Bisulfite-Conversion of DNA from Tissues, Cell Lines, FFPE Tissues, Aspirates, Lavages, Effusions, Plasma, Serum, and Urine. PLoS One. 2014;9(4):e93933. 10.1371/journal.pone.0093933 PMC3974851 24699908

[34] Marcil A, Dumontier E, Chamberland M, Camper SA, Drouin J (2003). Pitx1 and Pitx2 are required for development of hindlimb buds. Development.

[35] Takenobu M, Osaki M, Fujiwara K, Fukuhara T, Kitano H, Kugoh H (2016). PITX1 is a novel predictor of the response to chemotherapy in head and neck squamous cell carcinoma. Mol Clin Oncol.

[36] Dietrich D, Hasinger O, Liebenberg V, Field JK, Kristiansen G, Soltermann A (2012). DNA methylation of the homeobox genes PITX2 and SHOX2 predicts outcome in non-small-cell lung cancer patients. Diagn Mol Pathol.

[37] Laino L, Iezzi G, Piattelli A, Lo Muzio L, Cicciu M (2014). Vertical ridge augmentation of the atrophic posterior mandible with sandwich technique: bone block from the chin area versus corticocancellous bone block allograft—clinical and histological prospective randomized controlled study. Biomed Res Int.

[38] Cicciu M, Herford AS, Cicciu D, Tandon R, Maiorana C (2014). Recombinant human bone morphogenetic protein-2 promote and stabilize hard and soft tissue healing for large mandibular new bone reconstruction defects. J Craniofac Surg.

[39] Herford AS, Tandon R, Stevens TW, Stoffella E, Cicciu M (2013). Immediate distraction osteogenesis: the sandwich technique in combination with rhBMP-2 for anterior maxillary and mandibular defects. J Craniofac Surg.

[40] Lee Y, Kawagoe R, Sasai K, Li Y, Russell HR, Curran T (2007). Loss of suppressor-of-fused function promotes tumorigenesis. Oncogene.

[41] Dimitrova K, Stoehr M, Dehghani F, Dietz A, Wichmann G, Bertolini J (2013). Overexpression of the Hedgehog signalling pathway in head and neck squamous cell carcinoma. Onkologie.

[42] Zhang HZ, Shan CG, Huang AP, Wang JM. Characterization of gene methylation in human papillomavirus associated-head and neck squamous cell carcinoma. Genet Mol Res. 2016;15(3). https://www.ncbi.nlm.nih.gov/pubmed/27706614. 10.4238/gmr.15038206 27706614

[43] Lechner M, Fenton TR (2016). The genomics, epigenomics, and transcriptomics of HPV-associated oropharyngeal cancer—understanding the basis of a rapidly evolving disease. Adv Genet.

[44] Cheong A, Zhang X, Cheung YY, Tang WY, Chen J, Ye SH, et al. DNA methylome changes by estradiol benzoate and bisphenol A links early-life environmental exposures to prostate cancer risk. Epigenetics. 2016;11(9):674–89. 10.1080/15592294.2016.1208891 PMC5048723 27415467

[45] Marucci G, Fabbri PV, Morandi L, De Biase D, Di Oto E, Tallini G (2015). Pathological spectrum in recurrences of glioblastoma multiforme. Pathologica.

[46] Lin M, Smith LT, Smiraglia DJ, Kazhiyur-Mannar R, Lang JC, Schuller DE (2006). DNA copy number gains in head and neck squamous cell carcinoma. Oncogene.

[47] Schneider KU, Dietrich D, Fleischhacker M, Leschber G, Merk J, Schaper F (2011). Correlation of SHOX2 gene amplification and DNA methylation in lung cancer tumors. BMC Cancer.

